# Human amniotic membrane as newly identified source of amniotic fluid pulmonary surfactant

**DOI:** 10.1038/s41598-017-06402-w

**Published:** 2017-07-25

**Authors:** Angela Lemke, José Carlos Castillo-Sánchez, Florian Prodinger, Asja Ceranic, Simone Hennerbichler-Lugscheider, Jesús Pérez-Gil, Heinz Redl, Susanne Wolbank

**Affiliations:** 1 Ludwig Boltzmann Institute for Experimental and Clinical Traumatology / AUVA Research Center, Vienna, Austria; 2 Austrian Cluster for Tissue Regeneration, Vienna, Austria; 3 Departamento de Bioquimica, Facultad de Biologia, and Instituto de Investigación Hospital Doce de Octubre, Universidad Complutense, Madrid, Spain; 4 Department of Analytical Chemistry, Faculty of Chemistry, University of Vienna, Vienna, Austria; 5 Red Cross Blood Transfusion Service of Upper Austria, Linz, Austria

## Abstract

Pulmonary surfactant (PS) reduces surface tension at the air-liquid interface in the alveolar epithelium of the lung, which is required for breathing and for the pulmonary maturity of the developing foetus. However, the origin of PS had never been thoroughly investigated, although it was assumed to be secreted from the foetal developing lung. Human amniotic membrane (hAM), particularly its epithelial cell layer, composes the amniotic sac enclosing the amniotic fluid. In this study, we therefore aimed to investigate a potential contribution of the cellular components of the hAM to pulmonary surfactant found in amniotic fluid. We identified that cells within the native membrane contain lamellar bodies and express all four surfactant proteins as well as ABCA3. Lipidomic profiling by nanoESI – MS/MS revealed the presence of the essential lipid species as found in PS. Also, the biophysical activity of conditioned cell culture supernatant obtained from hAM was tested with captive bubble surfactometry. hAM supernatant showed the ability to reduce surface tension, similar to human PS obtained from bronchoalveolar lavage. This means that hAM produces the essential PS-associated components and can therefore contribute as second potential source of PS in amniotic fluid aside from the foetal lung.

## Introduction

Pulmonary surfactant (PS) is known to be produced and secreted by alveolar epithelial type II cells as a surface-active lipid-protein complex. Its function relies on minimizing surface tension at the air-liquid interface and therefore enabling the gas exchange in the lung by preventing the collapse of alveoli during expiration^1, 2^. The predominant component of PS are lipids (90%), mainly comprising dipalmitoylphosphatidylcholine (DPPC; 40%) and other phospholipids (40%). The latter ones include unsaturated PC species and around 15% of anionic phosphatidylglycerol (PG) and phosphatidylinositol (PI)^2, 3^. These lipids changed evolutionary to enhance surfactant activity^4^ and to enable extreme minimization of surface tension^5^. Only a minor part of PS is composed of plasma proteins (5%) and surfactant-associated proteins (SP-A, B, C and D, 5%), which are playing an important role irrespective of their minor presence^6^.

In general, if the function of PS is disturbed or if it is present at insufficient amounts, the consequences can be fatal. Surfactant deficiency can occur in babies from immature lung development during foetal growth, leading to infant respiratory distress syndrome (IRDS). This occurs in approximately one per cent of all newborns and is the leading cause of death in preterm babies^7, 8^. But also acquired PS failure, emerging from acute lung injury, can lead to acute respiratory distress syndrome (ARDS), chronic lung diseases and even lung failure, also in the adult^9^.

Therapeutic options to improve complications associated with these diseases are limited. One therapeutic strategy is the exogenous application of surfactant formulations, such as synthetically produced or animal-derived surfactants. This can increase the chances of survival^8, 10, 11^, although beneficial effects in ARDS are limited^12, 13^.

During foetal development, PS does not form until the end of gestation, which is the reason why insufficient surfactant is a major problem in preterm neonates^4^. An indication of the foetal pulmonary maturity is the examination of amniotic fluid for levels of surfactant^14, 15^. However, so far the origin of this surfactant had not been ultimately determined, though it was supposed to be secreted by the foetal developing lung^16^. The amniotic fluid is surrounded by the human amniotic membrane (hAM), the innermost of the foetal membranes. hAM is of foetal origin and is derived from the embryonic epiblast prior to gastrulation^17^. The cells of the hAM as well as the membrane itself exhibit immunoregulatory^18, 19^, anti-fibrotic^20, 21^, and non-tumorigenic^22, 23^ characteristics. Also, the membrane has for centuries been used as allograft because of its low immunogenicity^18, 23^ and beneficial effects in wound healing and corneal damage^22, 24^. Furthermore, hAM and cells thereof have proven stem cell characteristics and were successfully differentiated into cell types of all three germ layers^23, 25^.

Since hAM and especially amniotic epithelial cells directly envelop the amniotic fluid, we investigated hAM isolated from term placenta including cells derived thereof for the expression and secretion of PS components. This would indicate a potential contribution of hAM to the surfactant found in the amniotic fluid.

## Material and Methods

### Preparation and cultivation of hAM

Human placentae were obtained at term pregnancy during Caesarean sections with written and informed maternal consent and collection approved by the local ethical board (Ethikkommission des Landes Oberösterreich). All methods were performed according to the relevant guidelines and regulations of the local ethical board. Until processing, placentae were stored in sterile bags (Websinger, Austria) in Ringer solution (Fresenius, Austria).

hAM was peeled off by blunt dissection and separated into the placental and reflected subregion, each comprising both epithelial and mesenchymal layer. The reflected region of hAM represents the part of the amniotic sac that is not attached to the placenta, while the placental hAM is loosely connected with the placenta^26^. After several washing steps in 1x phosphate buffered saline (PBS, Lonza, Switzerland), 8 mm biopsy punches (Kai medical, Germany) were taken. These were either directly processed for ultrastructural and (immuno)histological analysis or transferred into 96-round bottom well plates containing 200 µL culture medium (Dulbecco’s Modified Eagle’s Medium (DMEM) high glucose, 10% FBS, 200 mM glutamine, and 1% Penicillin-Streptomycin, all from Sigma-Aldrich, USA). Medium was changed every 2–3 days.

### Isolation and cultivation of human amnion-derived epithelial (hAE) and mesenchymal stromal (hAMS) cells

hAE cells were isolated according to a protocol similar to Stadler *et al*.^27^. Briefly, hAM (placental and reflected) was cut into small pieces (3–4 × 2 cm²) and placed into digestion solution. hAE cells were segregated from the basal membrane during 3 digestion steps, each comprising 10–20 min, using 0.05–0.1% trypsin/EDTA (Sigma-Aldrich, USA). Cell suspensions of the second and third digestion step were filtered through a 100 µm cell strainer (BD, Austria). Then they were centrifuged at 400 g for 9 min, and either directly fixed for flow cytometry or seeded at a density of 3 × 10^4^ cells/cm² onto gelatine-coated glass cover slips (Carl Roth, Germany) in 24-well culture plates containing cell culture medium for immunofluorescence staining. After reaching confluence of approximately 80%, the cells were analysed.

The other cell type found in hAM, hAMS cells, were isolated by shaking in 0.1% collagenase (Biochrome, Germany) in DMEM (low glucose, Sigma-Aldrich, USA) for 2 hours in an incubator. After filtration through a 100 µm cell strainer, the cell suspension was centrifuged at 400 g for 9 min, the cells counted and afterwards fixed for flow cytometer analysis or cultured on glass cover slips until immunofluorescence staining.

### Transmission electron microscopy

hAM explants directly taken after isolation were fixed at 4 °C for 2 hours in 2.5% glutaraldehyde in 0.1 mol/l sodium phosphate buffer, pH 7.4. The samples were then rinsed with the same buffer, postfixed in 2% osmium tetroxide in ddH_2_O, washed in ddH_2_O, dehydrated in a graded series of acetone, and embedded in Agar 100 resin. 70-nm sections were post-stained and examined with an FEI Morgagni 268D (FEI, Eindhoven, The Netherlands) operated at 80 kV. Images were acquired using an 11 megapixel Morada CCD camera (Olympus-SIS).

### Scanning electron microscopy

hAM explants were washed in 1x PBS, then fixed in 2.5% glutaraldehyde in PBS (AppliChem, Germany) for 60 min at room temperature and afterwards dehydrated in an ascending ethanol series. After incubating in an ascending hexamethyldisilazane (HMDS, Sigma-Aldrich, USA) series, the membranes were air dried under the fume hood overnight. Then the biopsies were mounted onto double-sided sticky tape on top of an aluminium stub and sputter coated with Pd-Au using a Polaron SC7620 sputter coater (Quorum Technologies Ltd, GB). Afterwards they were analysed using a JEOL JSM-6510 scanning electron microscope (Jeol GmbH, Germany).

### Nile red (NR) staining

NR specifically stains intracellular neutral lipids (450–500 nm excitation wavelength) and phospholipids (515–560 nm excitation wavelength). Stock solution of NR (1 mg/mL in EtOH absolute, Sigma-Aldrich, USA) was prepared and stored under light protection. Staining was performed on hAM explants prior fixed in 10% neutral buffered formalin (VWR, USA) for 30 min. Explants were incubated in NR staining solution for 15 min after dilution to a final concentration of 500 ng/mL and kept in PBS afterwards. Nuclei were counterstained with DAPI. Analysis was carried out with an Axio Observer.A1 microscope (Zeiss, Germany) immediately afterwards using the 450–500 nm and 515–560 band pass excitation filters to detect intracellular neutral and phospholipid droplets, respectively.

### Immunohistological staining of hAM explants

Fixed hAM explants were rinsed in water and dehydrated in an ascending alcohol series. The tissue was embedded in paraffin and 4 µm thick sections were sliced in a rotary microtome. Sections were dried at 40 °C overnight, deparaffinised in xylene and rehydrated in a graded series of alcohol. Immunohistochemistry against pro- and mature surfactant protein B (SP-B) and prosurfactant protein C (pro-SP-C) was performed and sections were stained in Lab Vision Autostainer 360 (Thermo Fisher Scientific, UK). In the case of pro-SP-C, antibody retrieval was performed for 20 min at 95 °C using HIER T-EDTA pH 9.0 buffer (Zytomed, Germany) and for SP-B proteinase K (DAKO, Denmark) was used for 8 min. Afterwards, sections were washed in Tris-buffered saline and Tween (TBS, Thermo Fisher Scientific, UK). In order to deactivate endogenous peroxidase, sections were treated with UltraVision™ Hydrogen Peroxide Block (Thermo Fisher Scientific, UK) for 10 min and rinsed with the same buffer again. Subsequently, sections were exposed to the primary antibody against SP-B (working dilution 1:200, Abcam, UK) and pro-SP-C (working dilution 1:500, Abcam, UK) for 1 hour at room temperature. Afterwards, sections were rinsed with TBS and incubated with EnVision™ HRP anti-rabbit detection system (DAKO, Denmark) as a secondary antibody for 30 min at room temperature. Visualization was performed with ImmPACT™ NovaRED™ peroxidase substrate kit (Vector Laboratories, USA) and sections were counterstained with haematoxylin. Finally, slides were dehydrated and mounted with permanent mounting medium.

### Flow cytometric analysis of hAM-derived cells

hAE and hAMS cells were fixed immediately after isolation with 1% formaldehyde in PBS for 10 min. After two washing steps in 1% BSA (Sigma Aldrich, USA) in PBS, cells were either prior permeabilised for 20 min in Permeabilization Buffer (Affymetrix, USA) for intracellular staining or directly incubated with the respective primary antibodies for 45 min. Cells were washed and incubated with polyclonal Alexa Fluor 488-conjugated goat anti-rabbit or Alexa Fluor RPE-conjugated goat anti-mouse IgG secondary antibodies (1:750, Thermo Fisher Scientific, USA) for 30 min. Isotype controls were incubated with rabbit and mouse IgG. After washing, cells were analysed on a flow cytometer (Cytoflex, Beckman Coulter, USA). SP-A, Oct-4 and pan-CK were obtained from Santa Cruz Biotechnology (USA) and were used in a dilution of 1:100, all the other antibodies (SP-B, pro-SP-C, ABCA3, SSEA-4) were purchased from Abcam (UK) and were diluted 1:250. Every incubation step was performed on ice.

### Immunofluorescence staining of hAM-derived cells

Cells were fixed in 10% neutral buffered formalin for 15 min (VWR, USA) after reaching approximately 80% confluence, washed in PBS and non-specific binding was blocked with Roti®-ImmunoBlock (Carl Roth, Germany) for 45 min at room temperature. Subsequently, cells were permeabilised and incubated with primary antibodies against SP-B (1:250, Abcam, UK) and pro-SP-C (1:250, Abcam, UK) over night at 4 °C. After washing, cells were incubated with Alexa Fluor-conjugated goat anti-rabbit IgG secondary antibodies (1:750, Thermo Fisher Scientific, USA) for 60 min. For negative controls, IgG isotypes were applied. After washing in PBS, nuclei were counterstained and mounted with DAPI-containing anti-fading mounting medium (Roti®-Mount Fluor Care DAPI, Carl Roth, Germany). Staining was analysed with an Axio Observer.A1 microscope (Zeiss, Germany).

### Quantification of SP-D by ELISA

Conditioned cell culture supernatant of hAM biopsy punches and isolated hAE cells was collected within the first week of culture (hAM) or after reaching 80% confluence (hAE cells). After three to four days without medium change, the supernatant was frozen and kept at −80 °C until analysis. Subsequent to centrifugation to remove cell debris, SP-D was measured by ELISA (Hycult Biotechnology, The Netherlands) following the manufacturer’s instructions. As a positive control, bronchoalveolar lavage fluid (BALF) from male pigs was collected similar to Gushima *et al*.^28^. Briefly, 15 mL of sterile PBS was infused into each lung and gently aspirated. Afterwards the fluid was sterile filtered and centrifuged at 200 g for 10 min to remove debris and mucus clumps. BALF was stored at −80 °C until analysis and diluted 1:5 in PBS for ELISA.

### Extraction of lipids for mass spectrometric (MS) analysis

Cell culture supernatant of hAM explants was withdrawn and stored as described above. Extraction of lipids from hAM-derived supernatant and porcine BALF was carried out by liquid-liquid extraction using methyl-tert-butyl ether (MTBE) according to Matyash *et al*.^29^. For this, 400 µL of sample was mixed with 320 µL of methanol (VWR, USA) and 1 mL of MTBE (Honeywell Specialty Chemicals Seelze GmbH, Germany) and shaken for 1 h at 24 °C and 180 g. 250 µL of MS-grade water (Merck Millipore, USA) was added afterwards to induce phase separation. Following 10 min shaking at 24 °C and 180 g, centrifugation at 1500 g for 1 min was applied and the top organic layer carefully collected. Samples were then stored at −20 °C until MS analysis.

### Qualitative lipid profiling using shotgun MS

The MTBE extracts derived from hAM supernatants were measured using a Triversa Nanomate (Advion, USA) coupled to a LTQ Velos Orbitrap mass spectrometer (Thermo Fisher Scientific, USA). 8 μL of sample were mixed with 80 μL of measurement mix (70% (v/v) methanol, 30% (v/v) chloroform, 7.5 mM ammonium acetate); chloroform and ammonium acetate were purchased from Sigma-Aldrich, USA. Triversa Nanomate settings included 0.3 psi as backpressure and 1.4 kV spray voltage with a pick-up volume of 5 µL. The mass spectrometric analysis was conducted in the negative ion mode with an m/z window from 350–1,000 and normalised collision energy applied for fragmentation of 35. Data-dependent acquisition was chosen along with an isolation width for the precursor ion of 1 m/z and a dynamic exclusion m/z width of previously fragmented peaks of 0.5 m/z. Only singly charged ions were allowed for fragmentation, which was accomplished by collision induced dissociation (CID). Porcine BALF was used as a positive control and for comparison. Data analysis of the acquired raw files was carried out applying the free software LipidXplorer (Version 1.2.7).

### Quantitative analysis of lipids with supercritical fluid chromatography coupled with tandem mass spectrometry (SFC-MS/MS)

The SFC analyses were carried out using the Agilent 1260 SFC System directly coupled to the electrospray ionisation (ESI) source followed by a 6490 triple quadrupole mass spectrometer (Agilent Technologies, USA). The separation was performed on a 150 × 3 mm Luna HILIC separation column (Phenomenex, UK) employing a flow rate of 2 mL/min. Gradient mode was used for elution of the phospholipids. As mobile phase A supercritical carbon dioxide (Messer, Austria) and as mobile phase B methanol with 0.1% formic acid and 0.05% ammonium formate (both from Sigma-Aldrich, USA) were used. The gradient was started at 5% mobile phase B and increased to 40% B in 6 min. Mobile phase constitution was kept constant at 40% B for 1 min and reduced to 5% at 7.1 min. The chromatographic run was finished after a total measurement time of 9 min. The backpressure was set to 150 bar and the column temperature to 40 °C. For the targeted mass spectrometric analysis of DPPC the multiple reaction monitoring (MRM) transitions and the collision energies were optimized during the experiments. For the final analysis as quantifier the transition from m/z 734.4 to 184.1 occurring from the head group was used and as qualifiers m/z 734.4 to 86.1 (N,N,N-trimethyletheneaminium), m/z 734.4 to 478.32 (cleavage from the SN2 and H_2_O) and m/z 734.4 to 551.3 (cleavage from the SN1). For all transitions, the collision energies of 40 eV were used. Positive ionization mode was applied and the ESI spray voltage was 4000 V, gas flow 11 L/min, sheath gas temperature 400 °C, sheath gas flow 12 L/min, gas temperature 250 °C, and nebulizer gas pressure 35 psi. For quantification, an external calibration was performed.

### Biophysical activity assay: Captive Bubble Surfactometer (CBS)

Supernatants of placental and reflected hAM explants were collected as described above to isolate PS-like complexes. Samples were concentrated by consecutive ultracentrifugation at 120,000 g until getting a phospholipid concentration of 10 mg/mL, quantified using a commercial kit (Spinreact, Spain). Then, surface activity of the different surfactant samples were tested using the CBS^30^.

The assay is based on introducing a small amount of surfactant sample (typically 0.5 µl) below the surface of an air bubble floating against an agarose cap located in a chamber containing a buffer solution (Tris 5 mM, NaCl 150 mM, 10% sucrose, pH 7) (Supplementary Fig. S1). The agarose cap is attached to a piston, which allows performing compression-expansion cycles into the chamber. The resulting changes in surface tension can be deduced from the changes in bubble shape (Supplementary Fig. S1). First, the adsorption of a surfactant added to a clean air-liquid interface is recorded for 5 min. Then, the chamber is closed and the bubble is subjected to a quick expansion to register post-expansion adsorption for 5 min. Finally, the bubble is exposed to consecutive slow (quasi-static cycles) or fast (dynamic cycles) compression-expansion cycles (at 20 cycles/min) mimicking respiratory dynamics in the lung. The different parts of the CBS experiment are recorded with a camera focusing the bubble throughout the experiment. Surface tension values are calculated from bubble dimensions with a Data Analyses Software, according to a model published elsewhere^31^. PS isolated from BALF of porcine lungs (native surfactant, NS) was used as a reference of an optimal surface active agent.

### Evaluation and statistical analysis

ELISA results and flow cytometric analysis as well as DPPC quantification were expressed as mean and S.D.

### Data availability

The data generated during and/or analysed during the current study are available from the corresponding author on reasonable request.

## Results

### Amniotic epithelium ultrastructure differs between placental and reflected region

**Figure 1:**
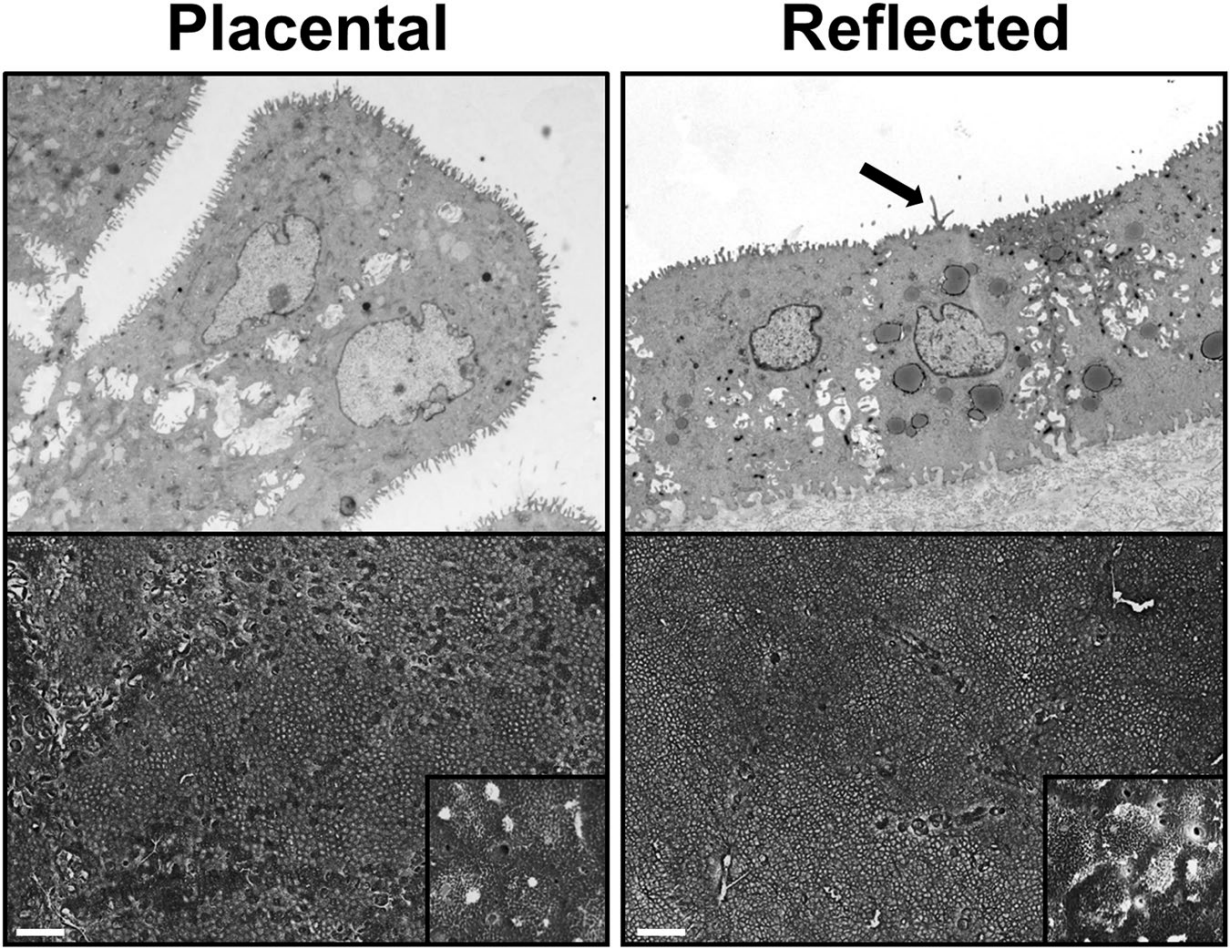
Ultrastructure of hAM differs between placental and reflected region. Epithelial cells of placental hAM display predominantly a columnar appearance covered with microvilli. Gaps between the cells are visible. Reflected hAM-derived epithelial cells are cuboidal and exhibit regular cell morphology, microvilli and longer cell protrusions (arrow) and are present at a high density covering the basal membrane. In this subregion of hAM, secretory vesicles are abundant. Upper row: Representative TEM pictures of amnion-derived epithelial cells, 3500x magnification; lower row: Representative SEM pictures of epithelial side of hAM, scale bars: 100 µm.

The ultrastructure of the epithelial layer of hAM from term placenta separated into placental and reflected region was analysed by transmission (TEM) and scanning electron microscopy (SEM) (Fig. 1). hAE cells from both subregions showed cell membrane protrusions covering the apical surface resembling microvilli, which are generally associated with secretion and typically found on PS-secreting pulmonary epithelial cells^32^. In general, the majority of cells from the placental region exhibited a columnar phenotype and a lower cell frequency on the membrane surface. In comparison, cells from the reflected area showed a dense and regular morphology, cuboidal appearance and a considerable amount of secretory vesicles inside the cytoplasm, indicating a strong secretory nature of the cells.

### Distinct expression of PS-associated components in hAM explants

**Figure 2:**
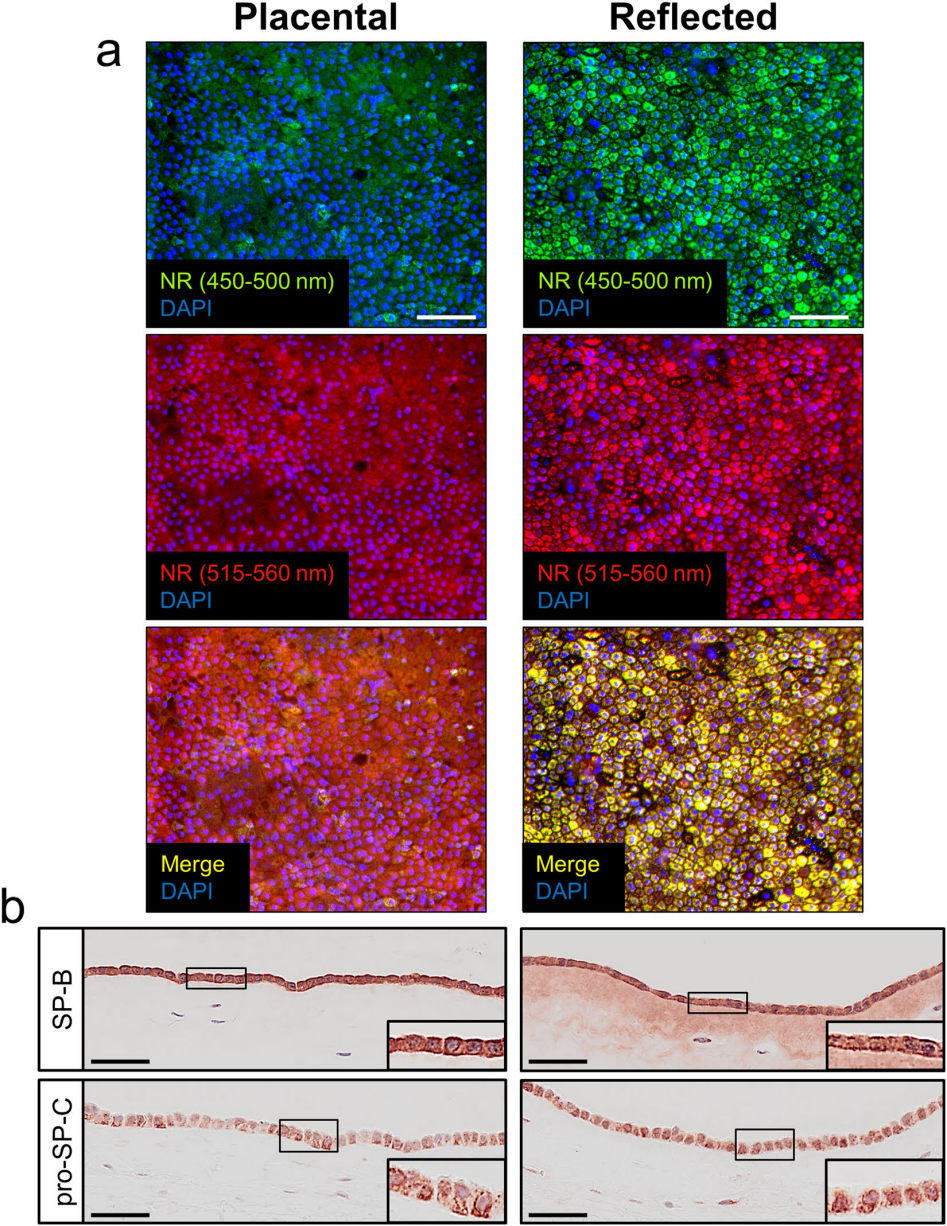
hAM explants show distinct expression of PS-associated components. (**a**) Visualization of intracellular (phospho-)lipid vesicles with NR staining. Explants of placental hAM display moderate numbers of neutral (upper row, green) and phospholipid vesicles (middle row, red), whereas cells of reflected hAM contain a considerable amount of both lipid classes covering the whole amniotic epithelium. n = 6. Upper row: neutral lipid vesicles (green, 450–500 nm excitation); middle row: phospholipid vesicles (red, 515–560 nm excitation); lower row: merge; counterstaining was performed with DAPI (blue). Scale bars: 50 µm. NR = Nile red. (**b**) Representative anti-SP-B and anti-pro-SP-C staining of hAM explants immediately after isolation. Placental-associated hAM shows strong SP-B expression (n = 4) in epithelial cells and fainter staining in mesenchymal cells, whereas in the reflected region of hAM even extracellular matrix exhibits specific SP-B staining. Pro-SP-C (n = 5) is similarly distributed in cells of both amniotic subregions. Scale bars: 50 µm.

To characterize hAM for intracellular lipid vesicles, explants from both amniotic regions were stained for lipid droplets with NR immediately after isolation. As shown in Fig. 2a, epithelial cells from the reflected area stained with great intensity also in the red spectrum suggesting a high content of phospholipid vesicles. hAE cells of the placental region were also homogenously stained, but at both wavelength ranges to a lower degree than cells of reflected amnion.

The next step was to identify specific PS protein components within the cells of hAM. For this, histological sections of hAM explants were stained for pro-SP-C and SP-B (Fig. 2b). The majority of epithelial cells of the reflected and the placental amnion exhibited a distinct intracellular staining for both surfactant proteins. Furthermore, the membrane of the reflected area demonstrated SP-B positive parts distributed in the extracellular matrix. In contrast, fewer (pro-SP-C) or hardly any (SP-B) of the hAMS cells expressed PS proteins in both hAM subregions (Supplementary Fig. S2).

### hAE cells are main source of PS proteins

**Figure 3:**
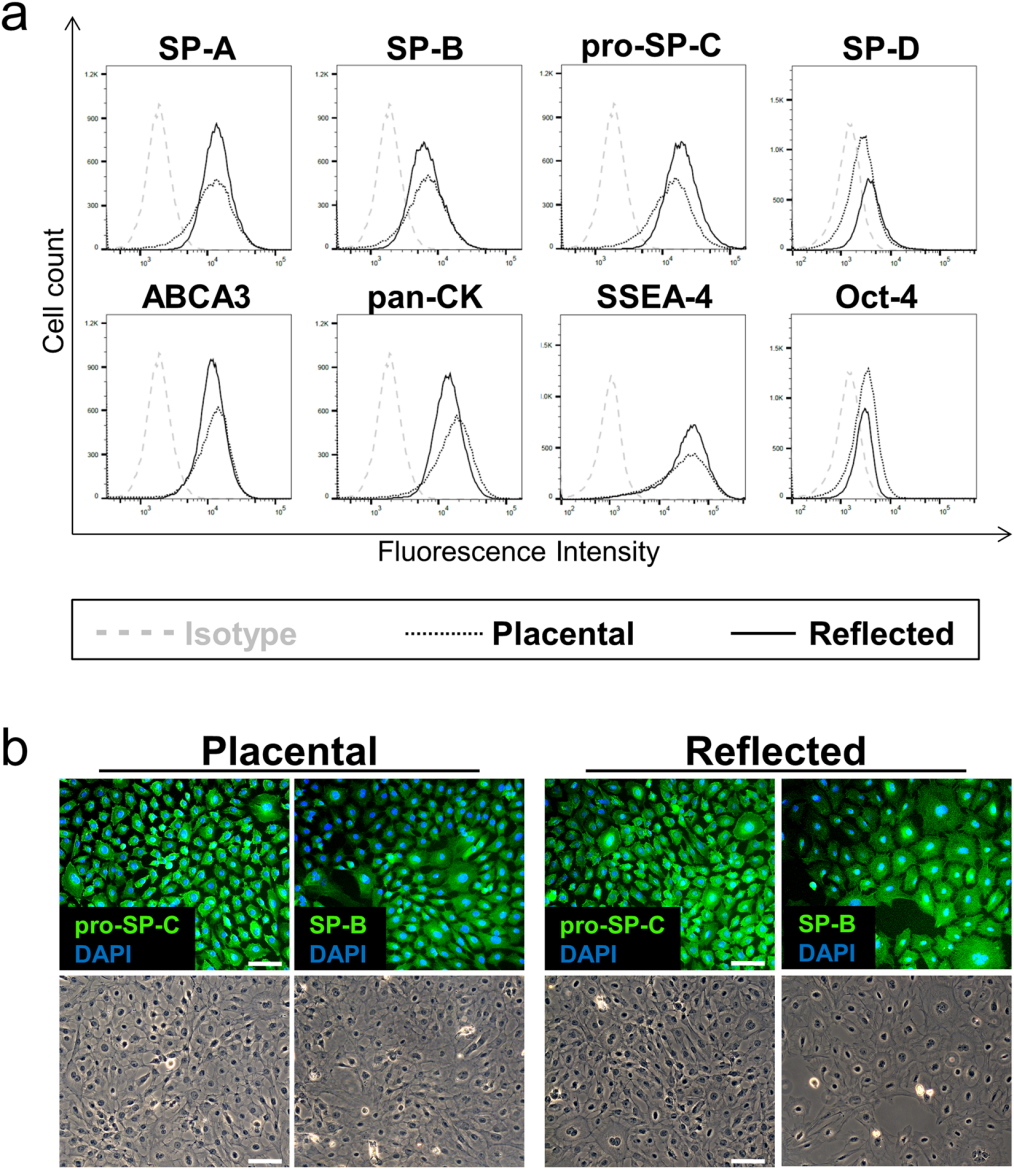
Expression of PS-associated proteins in isolated hAE cells. (**a**) Flow cytometric analysis of surfactant-associated proteins and stem cell markers immediately after hAE cells isolation. Cells show similar expression between both amniotic subregions, whereas in general hAE cells of the reflected region show moderately higher PS-associated protein expression and the placental area higher Oct-4 staining. n = 3–5. (**b**) Immunofluorescence staining of pro-SP-C and SP-B. The predominant part of hAM-derived epithelial cells exhibit distinct staining of both surfactant proteins in the cytoplasm, comparable in both amniotic subregions. Scale bars: 50 µm. n = 4.

In order to examine the cellular origin of PS proteins in hAM, hAE and hAMS cells were isolated from the two different subregions of the membrane and analysed for the production of surfactant proteins and ATP binding cassette subfamily A member 3 (ABCA3, Fig. 3a). This is a lamellar body membrane protein required for the formation of PS, which is expressed in alveolar epithelial type II cells. As summarized in Table 1, hAE cells analysed immediately after isolation via flow cytometry showed a high expression of three of the four surfactant proteins (SP-A, SP-B and pro SP-C) as well as ABCA3, whereas only a minor fraction of hAMS cells was positive for PS proteins (Supplementary Table S1). Fewer epithelial cells were positive for SP-D and almost none of the mesenchymal cells. In general, a higher percentage of hAE cells derived from reflected hAM expressed the surfactant-associated proteins, whereas cells from placental amnion showed higher percentages of cells positive for Oct-4 (8.1% ± 7.8 vs. 3.3% ± 2.8), a marker expressed in pluripotent stem cells. Almost all of the hAM-derived epithelial cells of both subregions expressed stage-specific embryonic antigen-4 (SSEA-4), in contrast to the mesenchymal cells, of which approximately half of the population was positively stained. The expression of SPs was further confirmed via immunofluorescent staining of cultured hAM-derived cells (Fig. 3b, Supplementary Fig. S3).

**Table 1:** Flow cytometric analysis of hAE cells immediately after isolation: Percentages of hAE cells isolated from the placental and reflected hAM region, positive for the respective marker.

Marker	Placental	Reflected	n
SSEA-4	95.4 ± 2.5	96.6 ± 1.1	3
Oct-4	8.1 ± 7.8	3.3 ± 2.8	5
SP-A	87.0 ± 4.8	91.7 ± 7.6	4
SP-B	67.1 ± 18.0	74.4 ± 19.2	4
pro-SP-C	89.0 ± 11.2	93.2 ± 7.0	4
SP-D	15.6 ± 8.4	28.7 ± 12.7	4
ABCA3	82.8 ± 11.4	90.8 ± 5.5	3

Illustrated are mean ± S.D. of given biological replicates.

### SP-D is actively secreted by hAM

**Figure 4:**
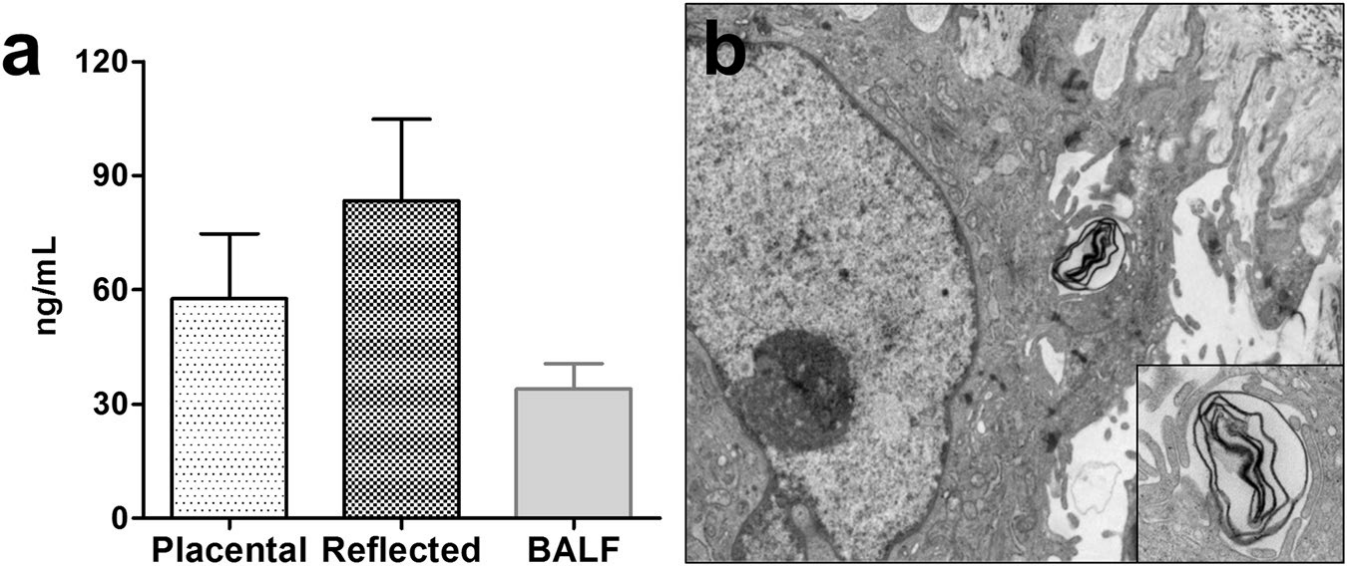
hAM explants actively secrete PS components. (**a**) SP-D is secreted into cell culture medium by hAM. Conditioned medium of reflected hAM explants shows higher SP-D concentrations (83.4 ± 21.5 ng/mL, n = 5) compared to placental hAM (57.8 ± 16.9 ng/mL, n = 5). As positive control, porcine BALF (34.0 ± 6.7 ng/mL, n = 2) was used. Illustrated are mean + S.D. (**b**) Ultrastructural analysis of hAM reveals lamellar body-like structures inside the cytoplasm, crucial for PS transportation and secretion. Magnification: 8900x and 18000x.

To confirm the potential of hAM to actively secrete PS-associated proteins, the conditioned supernatant of both isolated placental and reflected hAM explants was examined for SP-D by ELISA and compared with porcine BALF (Fig. 4a). SP-D was detectable in the supernatant of both subregions, but at considerably higher concentrations in reflected hAM-derived medium. Notably, SP-D concentration decreased moderately over time (Supplementary Fig. S4a), but was still detectable after a three-week culture period and was also secreted from isolated and cultured hAE cells (Supplementary Fig. S4b). The expression of surfactant proteins was sustained in hAE cells over several passages (Supplementary Fig. S4c). Moreover, ultrastructural analysis of these cells exhibited lamellar granular vesicles, strongly resembling lamellar bodies, which are crucial in storage and secretion of PS in the lung^33^ (Fig. 4b).

### hAM secretome exhibits main PS-associated lipids

**Figure 5:**
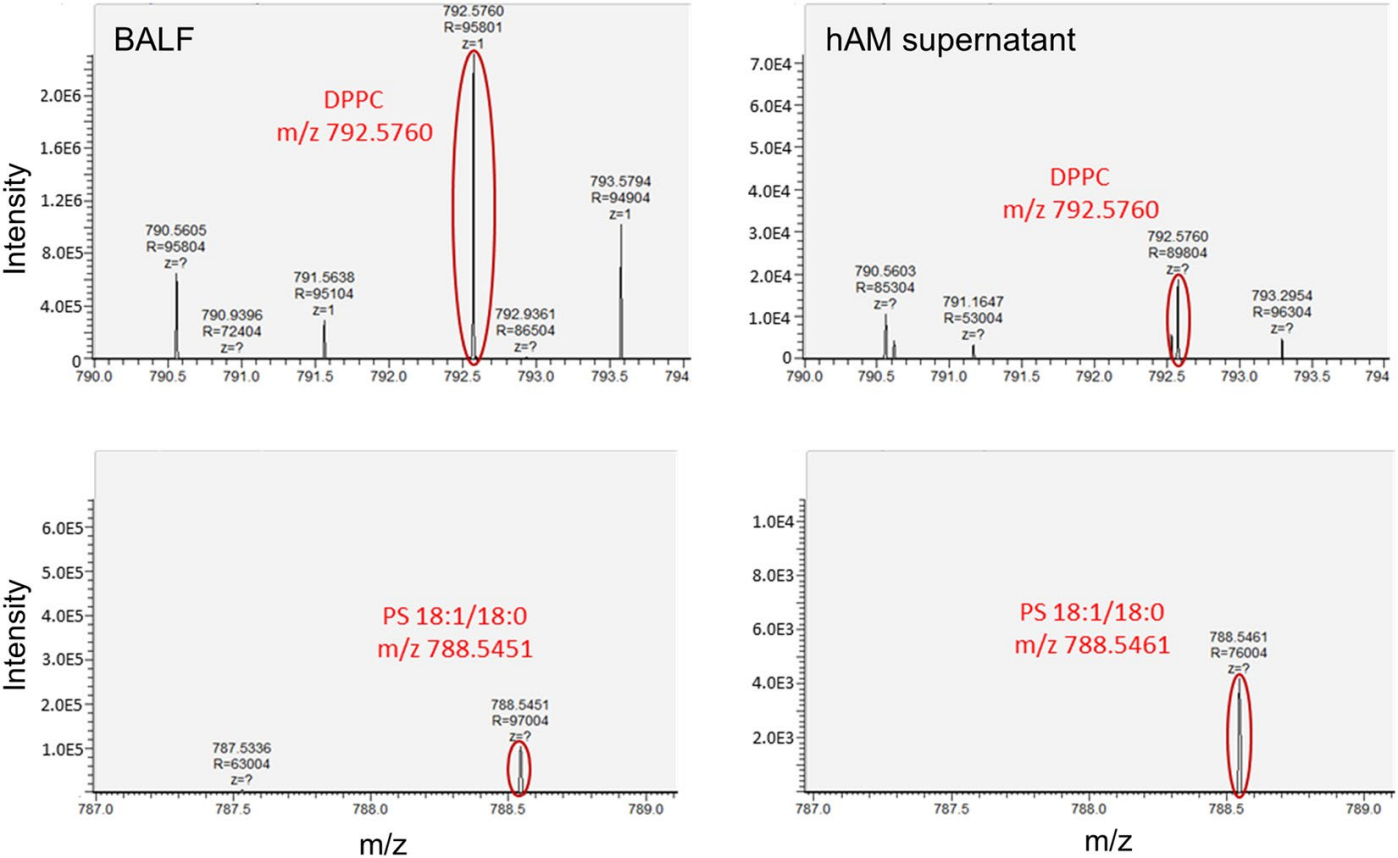
Lipidome analysis of hAM supernatant. Comparison of lipid species between porcine BALF (left) and hAM secretome (right) reveals secretion of the main PS-associated lipids by both subregions of hAM. Among others, dipalmityolphosphatidylcholine (DPPC, top) and phosphatidylserine (PS [18:1/18:0]), bottom) were detected. Illustrated are representative mass spectra supernatants of porcine BALF (left) and hAM supernatant (right) as analysed by nanoESI-MS/MS.

Four individual lipid species were identified in supernatants of explants of both amniotic subregions and of porcine BALF, namely the two ethanolamine plasmalogens PE-O [16:1/20:4] and PE-O [18:1/20:4], phosphatidylcholine (PC [16:0/16:0]) and phosphatidylserine (PS [18:1/18:0]). PC [16:0/16:0], also referred to as DPPC, is the most important phospholipid species for biophysical function of PS^5^. While a plethora of other signals associated with lipids were detected on the MS1 level, these four could also be identified on the MS2 level with satisfactory confidence (Fig. 5).

### Quantitative DPPC analysis of hAM-derived supernatants

**Figure 6:**
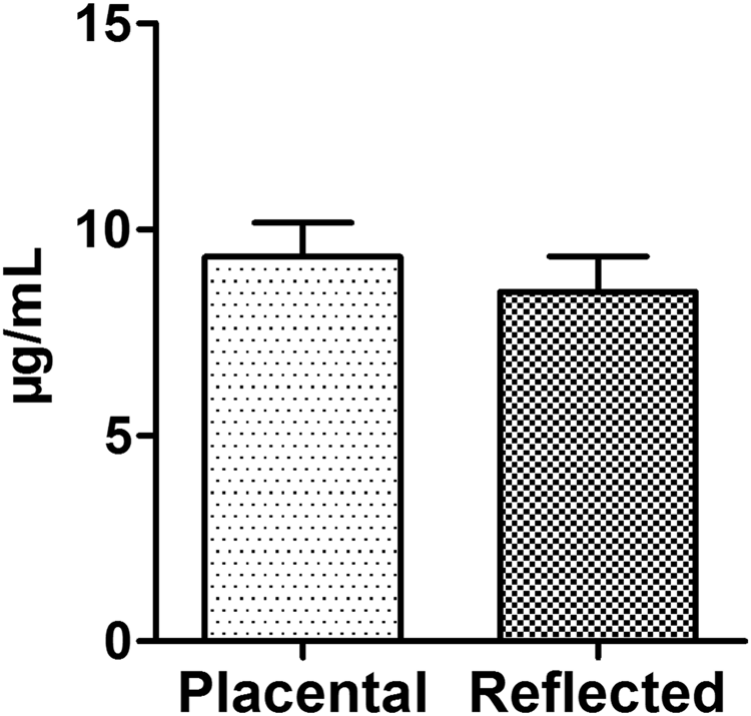
Evaluation of DPPC concentration in hAM-derived supernatants. Analysis of cell culture supernatant obtained from the two subregions of hAM revealed comparable amounts of DPPC, which is the predominant lipid in PS. Displayed are mean + S.D. Placental: n = 7; Reflected: n = 6.

DPPC as important player for surfactant functionality was examined on a quantitative level. Therefore, a targeted SFC-MS/MS method was developed for the exact quantitative analysis of DPPC. While DPPC could be found in all supernatants derived from hAM explants at concentrations ranging from 7.5 to 10.3 µg/mL, no differences in DPPC levels were detected between supernatants from the two distinct amniotic subregions (Fig. 6).

### PS-like material secreted by hAM shows limited functional activity

**Figure 7:**
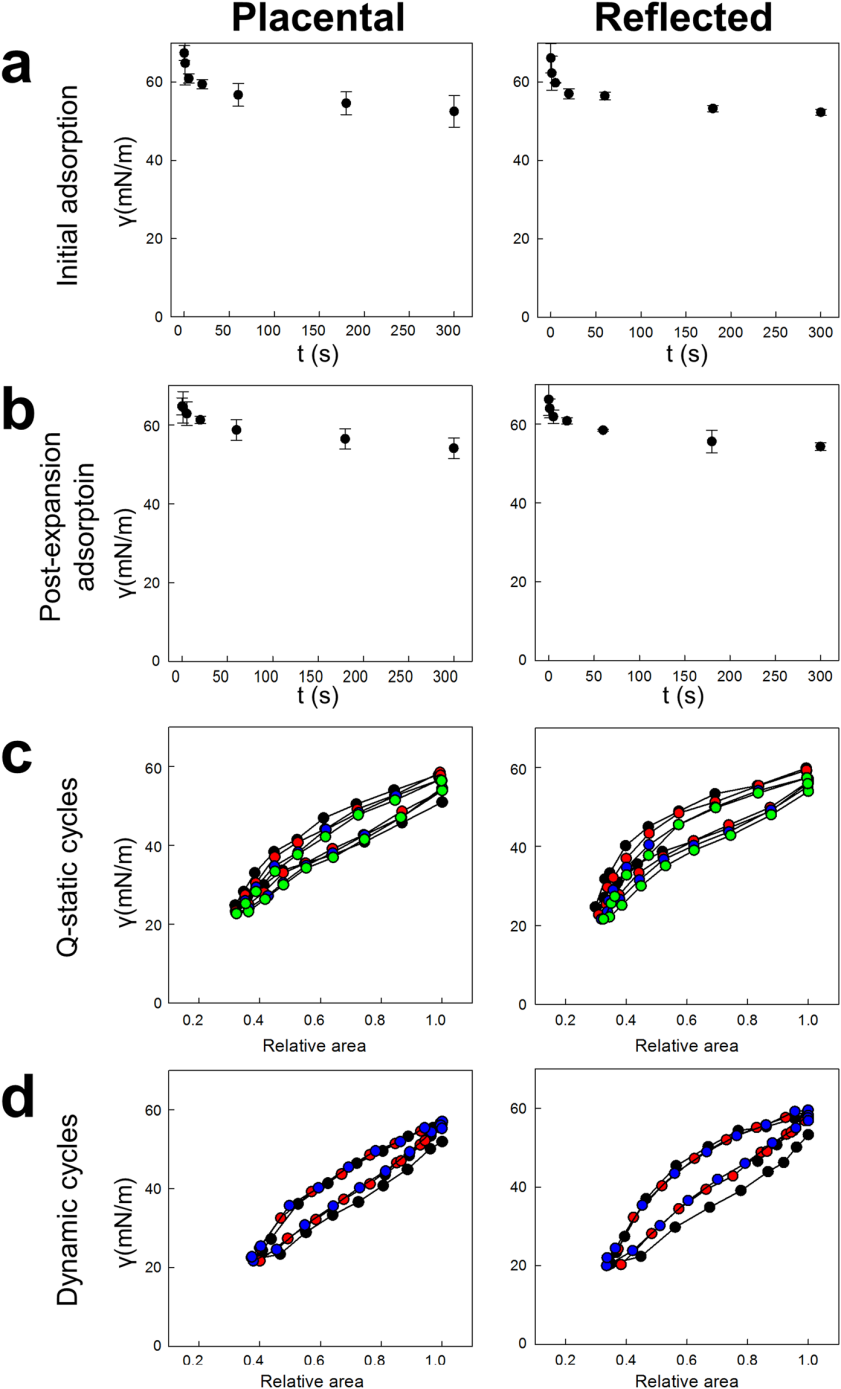
Biophysical behaviour of hAM supernatant tested using Captive Bubble Surfactometry. (**a**) Initial adsorption, (**b**) post-expansion adsorption and isotherms obtained during (**c**) slow (quasistatic) and (**d**) fast (dynamic) compression-expansion cycling of aqueous suspensions of PS-like material isolated from supernatants of placental and reflected hAM explants. Initial and post-expansion adsorptions are represented as average of three replicates. A representative replicate of three experiments is typified for quasistatic and dynamic cycles. All quasistatic cycles are illustrated (first in black, second in red, third in blue and fourth in green). Only first (black), tenth (red) and twentieth (blue) cycles are shown for dynamic cycles.

Lung surfactant is synthesized to reduce surface tension along respiratory cycles allowing respiratory dynamics and avoiding alveoli collapse. Because of that, biophysical properties of tissue culture supernatants derived from placental and reflected hAM explants were tested using CBS. Supernatants of both amniotic subregions did not reduce surface tension below 50 mN/m once injected to an air-liquid interface (Fig. 7a). This behaviour was not improved after subjecting the system to a quick expansion (Fig. 7b). However, compression of the surface films reduced surface tension down to around 20 mN/m, which required more than 60% area reduction, either under slow (quasistatic, Fig. 7c) or fast (dynamic “breathing-like”, Fig. 7d) compression-expansion cycles. Surface activity did not improve to yield lower activity after repetitive compression-expansion cycling of the films. No obvious differences in surface activity were observed between the supernatants of placental and reflected hAM.

## Discussion

hAM, as the innermost of the foetal membranes, encloses the growing foetus and the amniotic fluid, in which PS, consisting mainly of lipids and specific proteins, can be found as an indication of the foetal pulmonary maturity^14, 15^. As origin of this amniotic fluid-derived PS, the developing foetal lung has been reported but not been exclusively defined so far. We have therefore investigated hAM and cells thereof as potential additional source of PS, as the presence of the two hydrophilic proteins SP-A and SP-D in hAM was already described^34^. For these proteins expressed in hAM, an immunoregulatory and anti-pathogenic function has been proposed^35^, however no direct relation to the amniotic fluid PS.

We identified that cells within the native hAM of term placenta express all four surfactant-associated proteins in both amniotic subregions, which have crucial functions regarding surfactant: Aside from its biophysical properties lowering surface tension, surfactant also has an important function in pulmonary host defence mediated by SP-A and SP-D. These two hydrophilic proteins are involved in clearance of bacteria, fungi, dying cells and viruses through binding, thus initiating phagocytosis; also, they stimulate chemotaxis and have an anti-inflammatory effect^36, 37^. SP-B and SP-C are hydrophobic proteins required for the proper biophysical function and consequently indispensable for breathing^4, 38, 39^. Lack of functional SP-B, such as for genetic reasons, leads to respiratory failure unless lung transplantation is performed^40, 41^. SP-C deficiency is not as critical, but leads to a decreased stabilization of surfactant and affected patients usually develop severe and chronic lung diseases^42^.

SP-A, SP-B and SP-D are primarily produced by alveolar epithelial type II cells, and in lower amounts by other cell types of the distal lung, such as the Club cells^43, 44^. While SP-A and SP-D are also present in other organs^45, 46^, SP-C is found exclusively in surfactant-producing cells^47^, which is a clear indication of a relation between hAM and the surfactant found in amniotic fluid. Moreover, we identified lamellar body-like structures, which are crucial for PS storage and secretion^48^ and the expression of ABCA3, required for the lamellar body biogenesis^49^, in amnion-derived cells. Also, secretory capability was demonstrated via ELISA, for which SP-D could be identified in hAM-derived cell culture supernatant over several weeks. These results show that foetus-derived PS proteins do not only adsorb on hAM, but demonstrates that they are actively produced and secreted by hAM cells.

The main part of PS consists of lipids; therefore, we analysed hAM-derived supernatant for lipids and compared it with porcine BALF. We identified four lipids in supernatant of both placental and reflected hAM explants, hence representing mostly the same lipid class constitution as BALF. The lipids included phosphatidylcholine, ethanolamine plasmalogen, sphingomyelin and phosphatidylserine, with the exception of phosphatidylglycerol, which was not identified in the hAM-derived surfactant-like material. Yet the phospholipid class, especially DPPC, is responsible for the extreme reduction of surface tension at the air-water interface^5^, a lipid that was definitely identified as major component in our hAM-derived supernatants. However, proper PS function is also conferred by their protein components^5^ rendering natural PS usually more efficient compared to most of the synthetically-produced ones^50^.

Native surfactant has the ability to extremely reduce surface tension at the air-liquid interface at the end of expiration in the lung^2, 3^. We tested the biophysical activity of our hAM-derived surfactant-like material with a captive bubble surfactometer. Although we could not reach the level of reduction of surface tension during initial and post-expansion adsorption as native surfactant^5^, we could demonstrate a certain functional activity of the hAM-derived surfactant-like material during quasistatic and dynamic cycling. It resembled the biophysical behaviour obtained with human non-bronchoscopic BALF^51^. This indicates at least some functional activity of the surfactant components secreted by hAM. One explanation for the lower biophysical activity of hAM-derived supernatant could be associated with the exposure of secreted surfactant to a medium enriched with FBS. Serum proteins, such as albumin, are potent PS inhibitors^52, 53^, which could have limited the functionality of the PS secreted by hAM. Moreover, the stimulation of secretion with β-adrenergic agents or secretagoges, such as ATP (adenosine triphosphate) and PMA (phorbol 12-mirystate 13-acetate) might be essential to induce substantial levels of PS secretion from hAM, as the PS exocytosis is tightly regulated^54^. This could also be the reason why we could not detect any significant differences in biophysical activity and DPPC concentration between the supernatants from the two subregions of hAM. Yet, a distinctly higher amount of lipid droplets was found in the cytoplasm of cells of reflected hAM compared to those of the placental region. Stimulation of hAM could increase secretion of lipid vesicles and consequently disclose a higher surfactant lipid concentration in reflected amnion-derived supernatant. This might then potentially lead to an improved PS function, for which a high phospholipid, most of all DPPC, concentration is required. Such experiments, including a direct extraction of PS from homogenized or secretion-stimulated hAM, could be part of future studies. Recently a difference in mitochondrial activity^55^ as well as in gene expression^26^ between the two subregions of hAM was demonstrated. Within this study, we identified an overall higher percentage of cells expressing the pluripotency marker Oct-4 in placental hAM-derived cells compared to those of the reflected area. Although not really relevant in regard to PS, this result might be interesting for differentiation and stem cell studies.

Cells isolated from hAM have previously been considered as a potential cell therapy for human lung diseases. For both hAE as well as mesenchymal decidual cells, the development of an alveolar epithelial cell phenotype, secreting PS proteins upon *in vitro* differentiation, has been reported^21, 54^. Moodley *et al*. did however not find these characteristics in undifferentiated hAE cells^21^. Moreover, the cells have proven promising in several pulmonary pathologic conditions *in vivo* without prior differentiation. They reduced inflammation and fibrosis and partially restored the physiological lung function after bleomycin-induced lung injury in mice^21, 56^ or after inflammation- or ventilation induced lung injury in sheep^57, 58^ and resulted in an engraftment into the lung in some cases^21, 58^. The reason for these beneficial effects in those studies could at least partially be hAM’s ability to produce surfactant-like material. *In vitro* differentiation of hAM or hAM-derived cells similar to Moodley *et al*.^21^ towards an alveolar epithelial cell type might be helpful and increase surfactant production. Both, isolated amniotic cells^59^, as well as the whole membrane^60, 61^, have already been transplanted in human patients for diverse therapeutic reasons, without eliciting major adverse immune reactions. Therefore, the application of hAM and hAM-derived cells is considered to be safe, despite an allogeneic usage^62^. Formulations derived from human amnion, such as a PS-enriched secretome, may represent a novel therapeutic approach for surfactant-related diseases. These could include respiratory distress syndrome, asthma or surfactant metabolism dysfunction, as potential therapeutic targets. However, substantial improvements, including an increase of PS concentration and a potential enhancement of the biophysical activity of hAM-derived PS, would be a prerequisite.

We showed that hAM-derived cells from term placenta already produce and secrete components similar to PS secreted by alveolar epithelial type II cells in the lung. All in all, this study demonstrates that hAM from term placenta has the potential to serve at least as a second source of PS in amniotic fluid aside from foetal lung. The functional relevance, such as a possible role of hAM in foetal lung maturation or in pulmonary host defence of the growing fetus, remains however to be determined.

## Electronic supplementary material

Supplementary Information

## References

[1] Schürch S, Goerke J, Clements JA (1976). Direct determination of surface tension in the lung. Proc. Natl. Acad. Sci. USA.

[2] Goerke J (1998). Pulmonary surfactant: functions and molecular composition. Biochim. Biophys. Acta - Mol. Basis Dis..

[3] Parra E, Pérez-Gil J (2015). Composition, structure and mechanical properties define performance of pulmonary surfactant membranes and films. Chem. Phys. Lipids.

[4] Orgeig S, Daniels CB, Johnston SD, Sullivan LC (2003). The pattern of surfactant cholesterol during vertebrate evolution and development: Does ontogeny recapitulate phylogeny?. Reproduction, Fertility and Development.

[5] Wüstneck R (2005). Interfacial properties of pulmonary surfactant layers. In Advances in Colloid and Interface Science.

[6] Serrano AG, Perez-Gil J (2006). Protein-lipid interactions and surface activity in the pulmonary surfactant system. Chemistry and physics of lipids.

[7] Zimmermann LJI, Janssen DJMT, Tibboel D, Hamvas A, Carnielli VP (2005). Surfactant metabolism in the neonate. Biol. Neonate.

[8] Merrill, J. D. & Ballard, R. a. Pulmonary surfactant for neonatal respiratory disorders. *Curr*. *Opin*. *Pediatr*. **15**, 149–54 (2003). 10.1097/00008480-200304000-00002 12640270

[9] Gregory TJ (1991). Surfactant chemical composition and biophysical activity in acute respiratory distress syndrome. J Clin Invest.

[10] Lewis JF, Veldhuizen R (2003). The role of exogenous surfactant in the treatment of acute lung injury. Annu. Rev. Physiol..

[11] Strohmaier, W., Redl, H. & Schlag, G. Studies of the Potential Role of a Semisynthetic Surfactant Preparation in an Experimental Aspiration Trauma in Rabbits. **16**, 101–110 (1990). 10.3109/01902149009087875 2328709

[12] Spragg RG (2011). Recombinant surfactant protein C-based surfactant for patients with severe direct lung injury. Am. J. Respir. Crit. Care Med..

[13] Kesecioglu J (2009). Exogenous natural surfactant for treatment of acute lung injury and the acute respiratory distress syndrome. Am. J. Respir. Crit. Care Med..

[14] Gluck L (1995). Diagnosis of the respiratory distress syndrome by amniocentesis. Am. J. Obstet. Gynecol..

[15] Autilio C (2017). A Noninvasive Surfactant Adsorption Test Predicting the Need for Surfactant Therapy in Preterm Infants Treated with Continuous Positive Airway Pressure. J. Pediatr..

[16] Blackett PR, McConathy WJ (1982). Comparison of lipids and apolipoproteins in amniotic fluid, neonatal urine, and cord serum. Ann. Clin. Lab. Sci..

[17] Cross JC (1998). Formation of the placenta and extraembryonic membranes. Ann. N. Y. Acad. Sci..

[18] Bailo M (2004). Engraftment potential of human amnion and chorion cells derived from term placenta. Transplantation.

[19] Wolbank S (2007). Dose-dependent immunomodulatory effect of human stem cells from amniotic membrane: a comparison with human mesenchymal stem cells from adipose tissue. Tissue Eng..

[20] Ricci E (2013). Anti-fibrotic effects of fresh and cryopreserved human amniotic membrane in a rat liver fibrosis model. Cell Tissue Bank..

[21] Moodley Y (2010). Human amnion epithelial cell transplantation abrogates lung fibrosis and augments repair. Am. J. Respir. Crit. Care Med..

[22] Insausti CL (2010). Amniotic membrane induces epithelialization in massive posttraumatic wounds. Wound Repair Regen..

[23] Ilancheran S (2007). Stem cells derived from human fetal membranes display multilineage differentiation potential. Biol. Reprod..

[24] Kim JS, Kim JC, Hahn TW, Park WC (2001). Amniotic membrane transplantation in infectious corneal ulcer. Cornea.

[25] Miki T, Lehmann T, Cai H, Stolz DB, Strom SC (2005). Stem cell characteristics of amniotic epithelial cells. Stem Cells.

[26] Han YM (2008). Region-Specific Gene Expression Profiling: Novel Evidence for Biological Heterogeneity of the Human Amnion. Biol. Reprod..

[27] Stadler G (2008). Phenotypic shift of human amniotic epithelial cells in culture is associated with reduced osteogenic differentiation *in vitro*. Cytotherapy.

[28] Gushima Y (2001). Expression of matrix metalloproteinases in pigs with hyperoxia-induced acute lung injury. Eur. Respir. J. Off. J. Eur. Soc. Clin. Respir. Physiol..

[29] Matyash V, Liebisch G, Kurzchalia TV, Shevchenko A, Schwudke D (2008). Lipid extraction by methyl-tert-butyl ether for high-throughput lipidomics. J. Lipid Res..

[30] Schürch D, Ospina OL, Cruz A, Pérez-Gil J (2010). Combined and independent action of proteins SP-B and SP-C in the surface behavior and mechanical stability of pulmonary surfactant films. Biophys. J..

[31] Schoel WM, Schürch S, Goerke J (1994). The captive bubble method for the evaluation of pulmonary surfactant: surface tension, area, and volume calculations. BBA - Gen. Subj..

[32] Wang J (2007). Differentiated human alveolar epithelial cells and reversibility of their phenotype *in vitro*. Am. J. Respir. Cell Mol. Biol..

[33] Stahlman, M. T., Gray, M. P., Falconieri, M. W., Whitsett, J. a. & Weaver, T. E. Lamellar body formation in normal and surfactant protein B-deficient fetal mice. *Lab*. *Invest*. **80**, 395–403 (2000). 10.1038/labinvest.3780044 10744075

[34] Miyamura K (1994). Surfactant proteins A (SP-A) and D (SP-D): Levels in human amniotic fluid and localization in the fetal membranes. Biochim. Biophys. Acta (BBA)/Lipids Lipid Metab..

[35] Wright JR (2005). Immunoregulatory functions of surfactant proteins. Nat. Rev. Immunol..

[36] Mason RJ, Greenhough TJ, Voelker DR (1998). Surfactant protein A and surfactant protein D in health and disease. Am. J. Physiol..

[37] Lawson PR, Reid KB (2000). The roles of surfactant proteins A and D in innate immunity. Immunol. Rev..

[38] Weaver TE (1998). Synthesis, processing and secretion of surfactant proteins B and C. Biochimica et Biophysica Acta - Molecular Basis of Disease.

[39] Cochrane CG, Revak SD (1991). Pulmonary surfactant protein B (SP-B): structure-function relationships. Science.

[40] Nogee LM (1994). A mutation in the surfactant protein B gene responsible for fatal neonatal respiratory disease in multiple kindreds. J Clin Invest.

[41] Palomar LM (2006). Long-term outcomes after infant lung transplantation for surfactant protein B deficiency related to other causes of respiratory failure. J. Pediatr..

[42] Nogee LM (2002). Mutations in the surfactant protein C gene associated with interstitial lung disease. In Chest.

[43] Voorhout WF (1992). Immunocytochemical localization of surfactant protein D (SP-D) in type II cells, Clara cells, and alveolar macrophages of rat lung. J. Histochem. Cytochem..

[44] Wong CJ, Akiyama J, Allen L, Hawgood S (1996). Localization and developmental expression of surfactant proteins D and A in the respiratory tract of the mouse. Pediatr Res.

[45] Fisher JH, Mason R (1995). Expression of pulmonary surfactant protein D in rat gastric mucosa. Am. J. Respir. Cell Mol. Biol..

[46] Paananen R, Sormunen R, Glumoff V, van Eijk M, Hallman M (2001). Surfactant proteins A and D in Eustachian tube epithelium. Am. J. Physiol. Lung Cell. Mol. Physiol..

[47] Weaver, T. E. & Whitsett, J. a. Function and regulation of expression of pulmonary surfactant-associated proteins. *Biochem*. *J*. **273**, 249–264 (1991). 10.1042/bj2730249 PMC1149839 1991023

[48] Haagsman HP, van Golde LMG (1991). Synthesis and assembly of lung surfactant. Annu. Rev. Physiol..

[49] Cheong N (2007). ABCA3 is critical for lamellar body biogenesis *in vivo*. J. Biol. Chem..

[50] Moya F, Maturana A (2007). Animal-Derived Surfactants Versus Past and Current Synthetic Surfactants: Current Status. Clinics in Perinatology.

[51] De Luca D (2013). Clinical and biological role of secretory phospholipase A2 in acute respiratory distress syndrome infants. Crit. Care.

[52] Taeusch HW, Bernardino de la Serna J, Perez-Gil J, Alonso C, Zasadzinski JA (2005). Inactivation of pulmonary surfactant due to serum-inhibited adsorption and reversal by hydrophilic polymers: experimental. Biophys. J..

[53] Gunasekara L, Schoel WM, Schürch S, Amrein MW (2008). A comparative study of mechanisms of surfactant inhibition. Biochim. Biophys. Acta.

[54] Cerrada, A. *et al*. Human decidua-derived mesenchymal stem cells differentiate into functional alveolar type II-like cells that synthesize and secrete pulmonary surfactant complexes. *PLoS One* **9** (2014). 10.1371/journal.pone.0110195 PMC4198213 25333871

[55] Banerjee A (2015). Different metabolic activity in placental and reflected regions of the human amniotic membrane. Placenta.

[56] Murphy S (2011). Human amnion epithelial cells prevent bleomycin-induced lung injury and preserve lung function. Cell Transplant..

[57] Vosdoganes, P. *et al*. Human amnion epithelial cells as a treatment for inflammation-induced fetal lung injury in sheep. *Am*. *J*. *Obstet*. *Gynecol*. **205**, doi:10.1016/j.ajog.2011.03.054 (2011). 10.1016/j.ajog.2011.03.054 21640967

[58] Hodges, R. J. *et al*. Human amnion epithelial cells reduce ventilation-induced preterm lung injury in fetal sheep. *Am*. *J*. *Obstet*. *Gynecol*. **206** (2012). 10.1016/j.ajog.2012.02.038 22542124

[59] Hemphill C, Stavoe K, Khalpey Z (2014). First in man: Amniotic stem cell injection promotes scar remodeling and healing processes in late-stage fibrosis. Int. J. Cardiol..

[60] Branski LK (2008). Amnion in the treatment of pediatric partial-thickness facial burns. Burns.

[61] Rommel, N. *et al*. Secondary correction of posttraumatic orbital wall adhesions by membranes laminated with amniotic membrane. *Br*. *J*. *Oral Maxillofac*. *Surg*. **51** (2013). 10.1016/j.bjoms.2013.01.016 23434269

[62] Silini AR, Cargnoni A, Magatti M, Pianta S, Parolini O (2015). The Long Path of Human Placenta, and Its Derivatives, in Regenerative Medicine. Front. Bioeng. Biotechnol..

