# Supplementary material for: Human amniotic membrane as newly identified source of amniotic fluid pulmonary surfactant
Source: Sci Rep. 2017 Jul 25;7:6406. doi: 10.1038/s41598-017-06402-w (PMC5527005; doi:10.1038/s41598-017-06402-w)
Supplement: Supplementary file 1 — Supplementary Information [file 41598_2017_6402_MOESM1_ESM.pdf]

Human amniotic membrane as newly identified source of amniotic fluid pulmonary surfactant

Angela Lemke, José Carlos Castillo-Sánchez, Florian Prodinger, Asja Ceranic, Simone Hennerbichler-Lugscheider, Jesús Pérez-Gil, Heinz Redl, Susanne Wolbank

SUPPLEMENTARY INFORMATION

Supplementary Figures

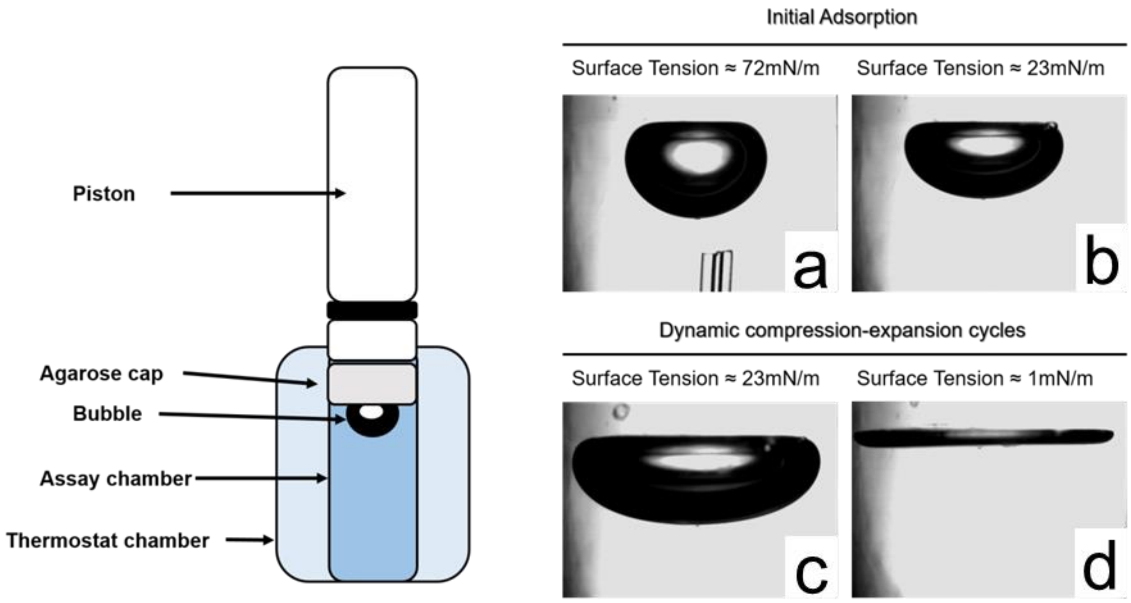

Supplementary Figure S1. Left) Schematic representation of the different parts of the CBS. A bubble is formed against an agarose cap attached to a piston allowing to compress and expand the bubble once the chamber is closed, to simulate respiratory mechanics. **Right) Surface tension changes produced by a porcine native surfactant sample used as a reference of an optimal surface active agent. (a)** Prior to surfactant adsorption, the surface tension of an air-liquid interface is 72mN/m. **(b)** After being adsorbed, native surfactant rapidly reaches equilibrium surface tension values of around 23mN/m. Next, re-spreading of surfactant complexes is monitored after a quick expansion of the bubble, recorded vs. time as post-expansion adsorption (not shown). **(c-d)** Finally, surface-active features of the surfactant film along compression-expansion cycles are tested. An efficient lung surfactant sample is able to reach minimal surface tension values at the end of compression (mimicking exhalation) from equilibrium values at the end of bubble expansion (as during inhalation).

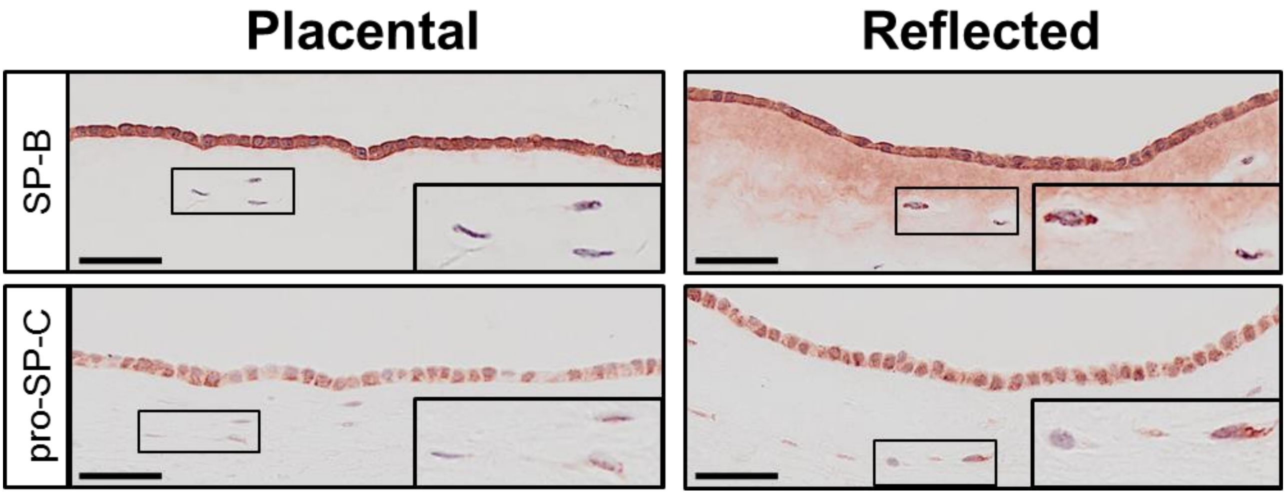

Supplementary Figure S2. Expression of SP-B and pro-SPC in hAM explants. hAMS cells of both subregions exhibit fainter staining of SP-B and pro-SPC antibodies in comparison to hAE cells. Displayed are representative sections of placental (left) and reflected (right) hAM explants immediately stained after isolation with anti-SP-B (n = 4) and anti-pro-SP-C (n = 5). Scale bars: 50 µm.

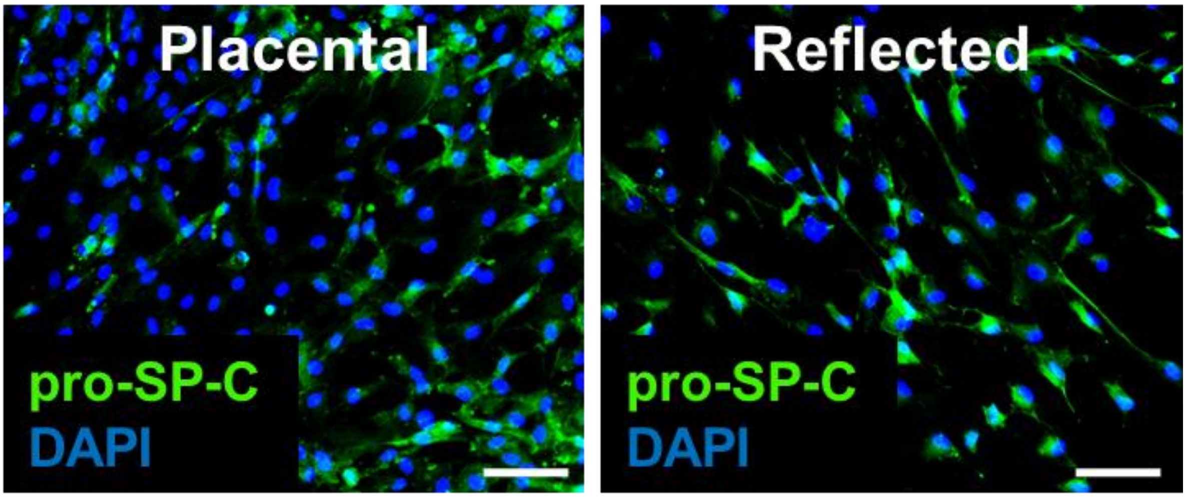

Supplementary Figure S3. Pro-SP-C expression in amnion-derived mesenchymal stromal (hAMS) cells. A certain amount of hAMS cells of both placental and reflected hAM express pro-SP-C in the cytoplasm after cell culture. Scale bars: 50 µm. n = 3.

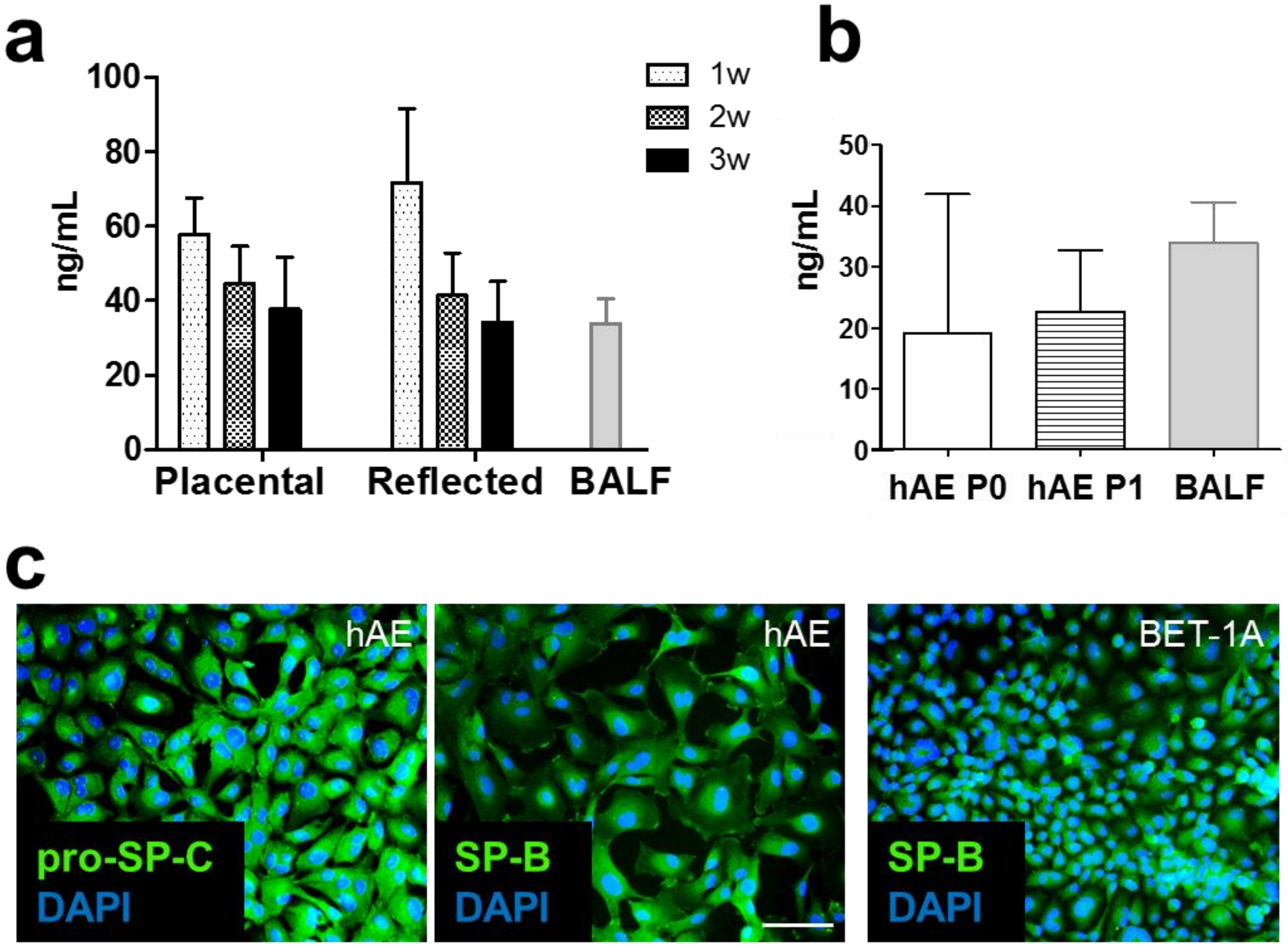

Supplementary Figure S4. Sustained expression of surfactant proteins. (a) Secretion of SP-D of hAM explants decreases moderately between one and three weeks of culture as determined by ELISA. n = 3; w = week. **(b)** SP-D secretion is maintained by hAM-derived epithelial cells at passage 0 (P0) and passage 1 (P1) after maintenance in culture medium. n = 4; BALF was used as positive control. **(c)** Cultured hAE cells preserve surfactant protein expression after passaging. Left: Pro-SP-C expression of hAE cells passage 1. Middle: SP-B expression of hAE cells passage 2. Right: SP-B expression of positive control BET-1A (immortalized human bronchial epithelial cells). Scale bar: 50 µm. n = 3-4.

Supplementary Table

Marker	Placental	Reflected	n
**SSEA-4**	48.9 ± 7.2	42.7 ± 15.5	3
**Oct-4**	0.4 ± 0.2	0.4 ± 0.0	3
**SP-A**	21.8 ± 36.9	33.8 ± 25.2	4
**SP-B**	13.4 ± 16.6	13.9 ± 15.0	4
**pro-SP-C**	39.8 ± 30.7	46.6 ± 22.5	4
**SP-D**	1.4 ± 1.0	0.9 ± 0.8	4
**ABCA-3**	40.2 ± 40.7	31.4 ± 20.1	3

Supplementary Table S1. Flow cytometric analysis of hAMS cells immediately after isolation: Percentages of hAMS cells isolated from the placental and reflected hAM region, positive for the respective marker. Illustrated are mean ± S.D. of given biological replicates.
